# The transcription factor *Nfix *is essential for normal brain development

**DOI:** 10.1186/1471-213X-8-52

**Published:** 2008-05-13

**Authors:** Christine E Campbell, Michael Piper, Céline Plachez, Yu-Ting Yeh, Joan S Baizer, Jason M Osinski, E David Litwack, Linda J Richards, Richard M Gronostajski

**Affiliations:** 1 Dept. of Biochemistry and New York State Center of Excellence in Bioinformatics and Life Sciences, State University of New York at Buffalo, 3435 Main St., Buffalo, NY 14214, USA; 2 Program in Neuroscience, State University of New York at Buffalo, 3435 Main St., Buffalo, NY 14214, USA; 3 Dept. of Physiology and Biophysics, State University of New York at Buffalo, 3435 Main St., Buffalo, NY 14214, USA; 4 Dept. of Anatomy and Neurobiology and the Program in Neuroscience, University of Maryland, Baltimore, School of Medicine, HSF II, S251, 20 Penn St., Baltimore, MD 21201, USA; 5 The School of Biomedical Sciences and The Queensland Brain Institute, The University of Queensland, Otto Hirschfeld Building, St Lucia, Queensland, 4072, Australia

## Abstract

**Background:**

The Nuclear Factor I (NFI) multi-gene family encodes site-specific transcription factors essential for the development of a number of organ systems. We showed previously that *Nfia*-deficient mice exhibit agenesis of the corpus callosum and other forebrain defects; *Nfib*-deficient mice have defects in lung maturation and show callosal agenesis and forebrain defects resembling those seen in *Nfia*-deficient animals, while *Nfic*-deficient mice have defects in tooth root formation. Recently the *Nfix *gene has been disrupted and these studies indicated that there were largely uncharacterized defects in brain and skeletal development in *Nfix*-deficient mice.

**Results:**

Here we show that disruption of *Nfix *by Cre-recombinase mediated excision of the 2nd exon results in defects in brain development that differ from those seen in *Nfia *and *Nfib *KO mice. In particular, complete callosal agenesis is not seen in *Nfix*-/- mice but rather there appears to be an overabundance of aberrant *Pax6*- and doublecortin-positive cells in the lateral ventricles of *Nfix*-/- mice, increased brain weight, expansion of the cingulate cortex and entire brain along the dorsal ventral axis, and aberrant formation of the hippocampus. On standard lab chow *Nfix*-/- animals show a decreased growth rate from ~P8 to P14, lose weight from ~P14 to P22 and die at ~P22. If their food is supplemented with a soft dough chow from P10, *Nfix*-/- animals show a lag in weight gain from P8 to P20 but then increase their growth rate. A fraction of the animals survive to adulthood and are fertile. The weight loss correlates with delayed eye and ear canal opening and suggests a delay in the development of several epithelial structures in *Nfix*-/- animals.

**Conclusion:**

These data show that *Nfix *is essential for normal brain development and may be required for neural stem cell homeostasis. The delays seen in eye and ear opening and the brain morphology defects appear independent of the nutritional deprivation, as rescue of perinatal lethality with soft dough does not eliminate these defects.

## Background

Nuclear Factor I (NFI) transcription/replication proteins are essential for both Adenoviral DNA replication [1-3] and for the regulation of transcription throughout development [4]. There are 4 NFI genes in mammals (*Nfia*, *Nfib*, *Nfic *and *Nfix*) and single NFI genes in *Drosophila*, *C. elegans*, *Anopheles*, Sea Urchin and other simple animals [4-6]. No NFI genes have been found in plants, bacteria or single cell eukaryotes. In mammals, NFI proteins form homo- or heterodimers and the 4 NFI genes are expressed in complex, overlapping patterns during embryogenesis [7,8]. NFI proteins bind to a dyad-symmetric binding site (TTGGCN_5_GCCAA) with high affinity [9,10], and NFI proteins have been shown to either activate or repress gene expression depending on the promoter and cellular context [4,11]. The presence of 4 NFI genes with possibly overlapping functions in mammals makes it a challenge to identify *in vivo *targets of individual NFI proteins and the roles of NFI genes in development.

Previous studies showed that NFI genes are essential for normal prenatal and postnatal mouse development. Disruption of *Nfia *causes multiple neuroanatomical defects including agenesis of the corpus callosum and hydrocephalus along with perinatal lethality [12]. More recent studies indicate that some of these brain defects may be due to altered formation of midline glial structures [13]. We also showed recently that NFIA and NFIB regulate oligodendrocyte differentiation in developing spinal cord [14] and that loss of *Nfia *can cause defects in kidney development [15]. Targeted disruption of *Nfib *also results in callosal agenesis and other neuroanatomical defects, but *Nfib*-/- animals die at birth, apparently due to a severe delay in lung maturation [16]. In contrast, loss of *Nfic *has a more modest phenotype with no observed brain defects but major defects in postnatal tooth development, including aberrant incisor formation and greatly reduced molar root formation [17]. *Nfic*-/- animals survive to adulthood and are fertile if their diet is supplemented with soft dough which presumably reduces the need for chewing. Here we have generated a conditional *Nfix *knockout (KO) mouse and analyzed the effect of germline loss of *Nfix*. As in *Nfia *and *Nfib *KO mice, loss of *Nfix *causes severe neuroanatomical defects including expansion of the cingulate cortex and the entire brain along the dorsal ventral axis, aberrant hippocampal development and the generation of excessive *Pax6*-expressing ventricular cells. These defects suggest that *Nfix *may function in the repression of neural stem cell proliferation or in cell migration. In addition, loss of *Nfix *causes delays in eye and ear opening and a decreased weight gain that appear related to the postnatal lethality seen in *Nfix*-/- mice.

## Results

### Growth phenotype of *Nfix*-/- animals

**Figure 1:**
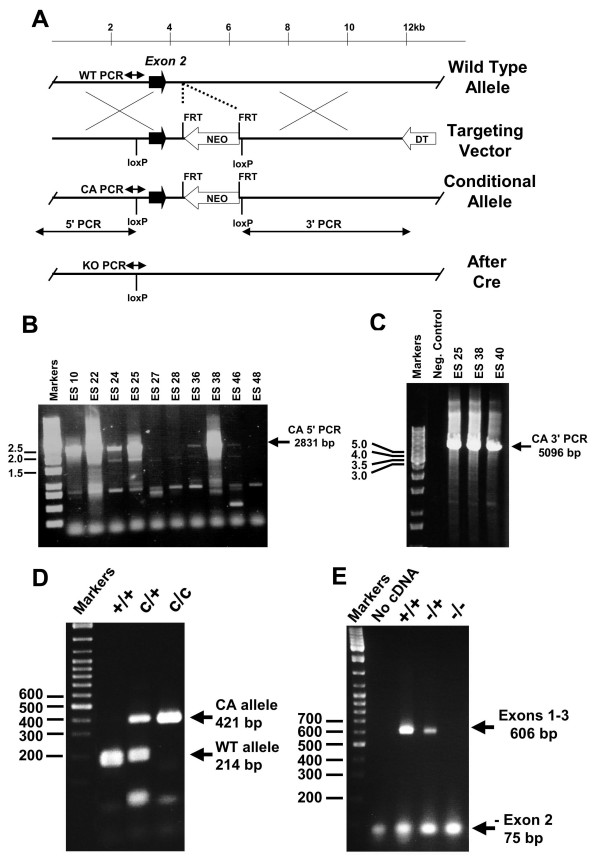
**Disruption of the *Nfix *gene**. **A) *Nfix *targeting vector construction and predicted PCR products**. Fragments including exon 2 of the wild type *Nfix *gene (Wild Type Allele) were used to construct the Targeting Vector. The Targeting Vector has one loxP site inserted ~400 nt 5' to exon 2 and a FRT-flanked PGK-Neo cassette (white arrow) with a second 3' loxP site ~600 nt 3' to the end of exon 2. Arrows show the location of PCR primers within genomic DNA used to detect the Wild Type Allele (WT PCR), the targeted Conditional Allele (CA PCR) and the Knockout allele (KO PCR); and primers from outside of and within the targeting vector used to verify correct genomic targeting at the 5' (5' PCR) and 3' (3' PCR) ends of the vector. Males with the Conditional Allele were bred with females expressing Cre recombinase in oocytes from the ZP3 promoter to generate the knockout allele (After Cre) lacking exon 2. **B&C) Nested PCR showing targeted integration at the 5' (B) and 3' (C) ends of the conditional allele**. **B) **A gel of products from nested PCR reactions with primers from genomic DNA 5' to the 5' end of the targeting vector, and primers within the 5'-most loxP site that yield no product from WT DNA and a 2831 bp product (arrow) from the conditional allele (CA 5' PCR). ES clones 10, 22, 24, 25 and 38 were strongly positive while clones 27, 28, 36, 46 and 48 were negative. Marker sizes in kb are on the left. Positive signals were verified in multiple PCR reactions. **C) **A gel of nested PCR products of primer pairs where one primer of each pair was located in genomic DNA outside of the 3'-most fragment of DNA present in the targeting vector and the other primer was present within the 3'-most loxP site of the targeting vector (3' PCR). Negative control DNA from non-electroporated ES cells shows no signal while clones 25, 38 and 40 yield a 5096 bp product (arrow, CA 3' PCR) specific for the correctly targeted Conditional Allele. The specificity of both the 5' and 3' PCR fragments was also confirmed by restriction enzyme digestion. Marker sizes in kb are on the left. **D) PCR genotyping of *Nfix *+/+, c/+ and c/c mice**. Mice heterozygous for the CA allele were bred, tail samples of progeny were collected, and the DNA was subjected to PCR using primers that flank the 5' most loxP site yielding products of 214 bp for the wild type (WT) allele and 415 bp for the Conditional (CA) Allele. Marker sizes in bp are on the left. **E) RT-PCR showing the absence of exon 2-containing *Nfix *transcripts in *Nfix*-/- mice**. RNA was prepared from the livers of *Nfix*+/+, -/+ and -/- animals, subjected to RT reactions and the resulting cDNA was subjected to PCR with primers in exons 1 and 3 of *Nfix*. RNAs containing exon 2 yield products of 606 bp while RNAs lacking exon 2 yield products of 75 bp. Note that some 75 bp product is formed from RNA in WT animals and we have confirmed by QPCR and sequencing that a small fraction of *Nfix *transcripts in WT animals are directly spliced from exon 1 to exon 3.

**Figure 2:**
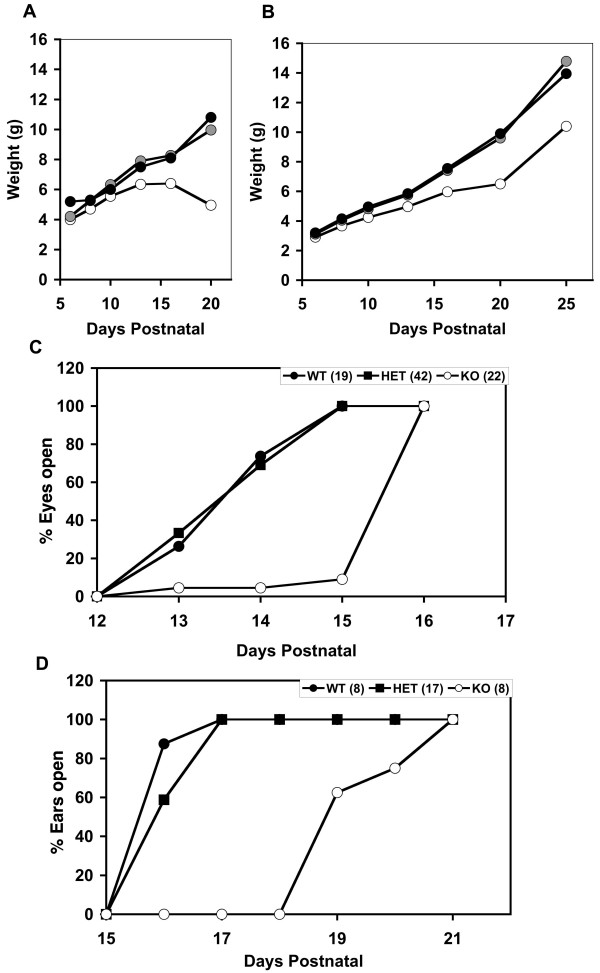
**Growth defects in *Nfix*-/- animals**. Delayed weight gain and development in *Nfix*-/- animals. **A) Reduced weight gain in *Nfix*-/- animals on regular chow**. The weights of progeny of *Nfix*-/+ parents were measured and plotted according to genotype. Note that *Nfix*-/- animals showed a slower weight gain from ~P6 and died at ~P22 when maintained on standard lab chow. Closed circles, WT (+/+); gray circles, *Nfix*-/+ (HET); open circles, *Nfix*-/- (KO). **B) Increased weight gain of *Nfix*-/- animals fed a soft dough diet**. The cages containing litters of *Nfix*-/+ parents were supplemented from P10 with soft transgenic dough. Weight gain and survival were increased substantially compared to non-supplemented litters (*e.g*. 2A). Symbols are as in panel A. **C) Delay in eyelid opening in *Nfix*-/- mice**. Eyelid opening was measured at various times after birth in progeny of *Nfix*-/+ animals. *Nfix*-/- animals showed an ~2 day delay in eyelid opening. Closed circles, WT (+/+); closed squares, HET (*Nfix*-/+); open circles, KO (*Nfix*-/-). The numbers following the genotype indicate the number of animals analyzed. **D) Delay in ear canal opening in *Nfix*-/- mice**. The opening of the ear canal was assessed at various times after birth in progeny of *Nfix*-/+ animals. *Nfix*-/- (KO) animals showed an ~4 day delay in ear canal opening. Symbols are as in panel C. The data in panels C and D are from an ~50% mixture of dough-supplemented and non-supplemented litters.

Initial breedings indicated that mice homozygous or heterozygous for the targeted Conditional Allele (Fig. 1A–D) had no obvious phenotypes (data not shown). Therefore the knockout allele (KO) was generated by Cre-mediated recombination and mice containing the KO allele were analyzed (Fig. 1E). *Nfix *transcripts extending from exon 1 to exon 3 were detected in both wild-type (WT) and heterozygous *Nfix*-/+ mice, but no intact transcripts were detected in *Nfix*-/- mice (Fig. 1E). Since exons 2 and 3 are in different protein reading frames, no functional proteins should be generated from transcripts lacking exon 2. Mice heterozygous for the knockout allele had no obvious phenotype and upon breeding their progeny were born at a Mendelian ratio (1:2:1,+/+:-/+:-/-, 52:127:51, 32 litters). However, by postnatal day 8 (P8) *Nfix*-/- (KO) animals began to gain less weight per day than their *Nfix *-/+ (HET) and +/+ (WT) littermates and by P14 the *Nfix*-/- animals began to lose weight (Fig. 2A). In addition, *Nfix*-/- animals opened their eyelids and developed open ear canals ~3 days later than +/+ or *Nfix*-/+ animals (Fig. 2C &2D), suggesting a general delay in epithelial development. In an effort to overcome the weight loss and lethality seen in the *Nfix*-/- animals, the diet of the *Nfix*-/- animals was supplemented with a soft dough feed that we showed previously could rescue lethality in *Nfic*-/- mice that have defects in tooth development. Surprisingly, while no tooth defects have been detected in the *Nfix*-/- animals, supplementation with soft dough increased their weight gain and they survived longer than animals fed a standard lab diet (Fig. 2B). The alleviation of some of the weight gain defect with soft dough addition indicates possible defects in the digestive tract, food sensing, or eating behavior in *Nfix*-/- animals.

### Aberrant brain development in *Nfix*-/- animals: Cortex and aberrant ventricular cells

**Figure 3:**
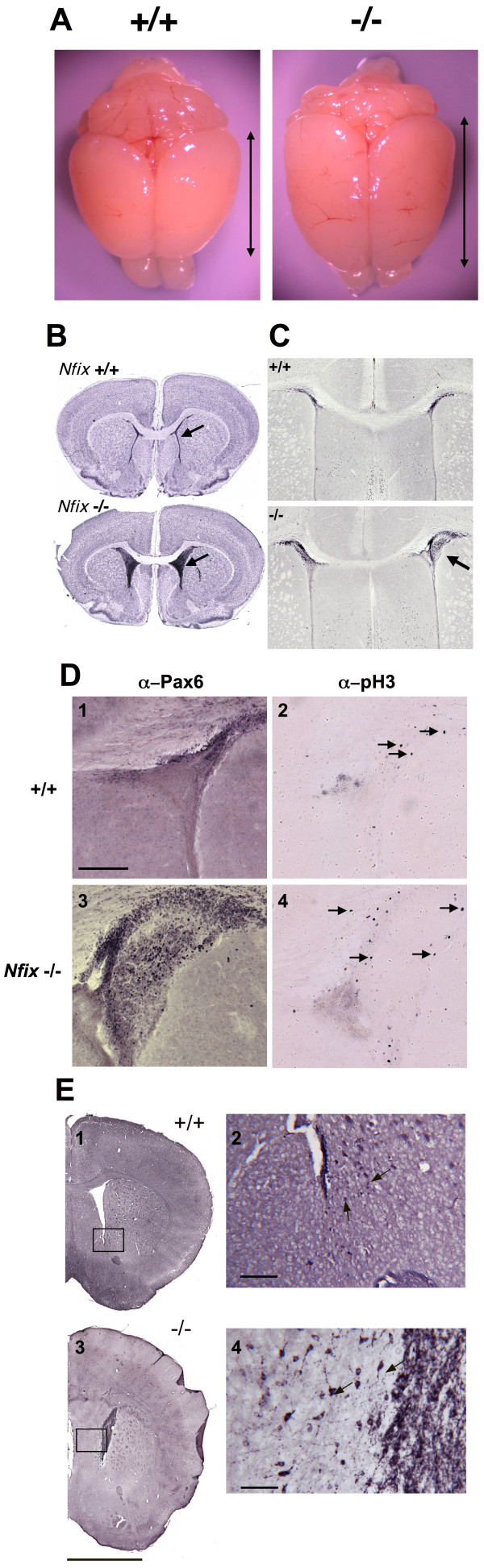
**Aberrant cortex depth and ventricular zone cells in *Nfix*-/- mice**. Brains from WT (+/+) and *Nfix*-/- animals were harvested, fixed in 4% PFA and imaged before **(A) **or after **(B-E) **sectioning, staining and mounting. **A) Gross structure of P22 brains of WT (+/+) and *Nfix*-/- animals**. Note difference in cortex anterior-posterior length (double-headed arrows). **B) Cresyl violet-stained coronal sections of P22 WT (+/+) and *Nfix*-/- brains**. Arrows denote normal ventricular region in *Nfix*+/+ and aberrant ventricular zone cells in *Nfix*-/- brains. **C) Immunostaining of *Pax6 *in coronal sections of P16 WT (+/+) and *Nfix*-/- brains**. Arrow shows *Pax6 *expression in aberrant ventricular zone cells of an *Nfix*-/- brain and in the ventricular zone of a +/+ littermate brain. **D)*Pax6 *and phospho-histone H3 staining of the ventricular region**. *Pax6 *(α-Pax6, panels 1&3) and phospho-histone H3 (α-pH3, panels 2&4) immuno-staining of ventricular regions of P16 WT (+/+, panels 1&2) and *Nfix*-/- (-/- panels 3&4) brains. Arrows in panels 2&4 denote some pH3-positive cells. Note that most of the *Pax6 *positive ventricular cells in the -/- brains do not react with α-pH3. Bar in panel 1 = 100 μm for D1-4 and 500 μm for both panels of C. **E) Doublecortin (DCX) immuno-staining in p69 WT (+/+) and *Nfix*-/- brains**. Panels 2&4 are higher magnification images of the boxed regions of panels 1&2, respectively. Arrows show the locations of some DCX-positive cells. Bars in panels 2&4 = 50 μm and in panel 3 = 2 mm.

We had shown previously that *Nfia*, *Nfib *and *Nfix *are all expressed in both prenatal and adult brains [8], and that loss of either *Nfia *or *Nfib *results in defects in brain development [12,13,16]. In addition, a previous description of an *Nfix*-/- mouse indicated some neuroanatomical defects [18]. We therefore examined the morphology of the brains of *Nfix*-/- animals. Our initial studies were performed on serial coronal sections of P22 brains of *Nfix*+/+ and *Nfix*-/- mice not maintained on supplemented diets. Gross examination of P22 *Nfix*-/- brains showed an apparent posterior extension of the cortical region that extended over the cerebellum (Fig. 3A, double arrows in -/- versus +/+). This was the first indication of abnormalities in the brains related to cortical development. In Fig. 3A the olfactory bulbs (OB) of the -/- animals appear somewhat smaller, but most or all of this size difference is due to accidental severing of portions of the bulb during brain removal. No major change in olfactory bulb size was seen in sections through the OB of 3 animals (not shown) but it is possible that subtle changes exist in some of the OB cell layers. We plan on examining OB structure in more detail in future studies. While WT and *Nfix*-/+ brains (not shown) exhibited the normal compact structure of the lateral ventricles (Fig. 3B, +/+ arrow), the ventricles of *Nfix*-/- mice were expanded and filled with cells of an unknown origin (Fig. 3B. -/- arrow). These cells stained darkly with Cresyl violet, were tightly packed, and serial sections showed that they filled the most anterior regions of the lateral ventricles at P22. Additional studies showed that these cells were also present in the ventricles of dough-supplemented animals (not shown) and all subsequent analyses were performed on diet-supplemented animals. Analysis of brains at different ages indicated that these aberrant cells were present as early as P0 and they remained until at least P69 (not shown). Since the cells filled regions of the lateral ventricles, we performed immuno-staining with markers previously shown to stain ventricular and subventricular cells. *Pax6 *is a transcription factor that is expressed at high levels in prenatal ventricular zone cells [19] and has previously been used as a marker for both prenatal and postnatal neural stem cells [20]. At P12–P16 (N = 3), *Pax6 *is highly expressed in both the normal ventricular zone cells in +/+ mice (Fig. 3C, +/+) and in the aberrant cells in the lateral ventricles of *Nfix*-/- mice (Fig. 3C, arrow -/-). The expression of this marker of neural progenitor cells suggests that these cells could represent an aberrant type of neural stem cell in the *Nfix*-/- animals. To determine whether any of these *Pax6 *positive cells were undergoing active proliferation we stained adjacent sections of brains with antibodies directed against *Pax6 *and phospho-histone H3 (pH3), a known marker of mitosis during cell proliferation. While 30–50% of the aberrant cells stained with α-*Pax6*, only a few cells in the ventricular zone of both +/+ and *Nfix*-/- mice stained with α-pH3 (Fig. 3D, arrows in panel 4). Thus at most only a small fraction of these aberrant cells are actively proliferating.

To assess whether some of the aberrant cells were early neuronal cells, sections of +/+ and *Nfix*-/- brains were stained with antibodies directed against doublecortin (DCX), a marker of migrating neurons [21]. A few DCX expressing cells are seen in the +/+ brain, possibly representing migrating neurons destined for the rostral migratory stream (Fig. 3E, panels 1–2, arrows) [21]. In contrast, 50–100% of the aberrant cells stained with α-DCX (Fig. 3E, panels 3–4), indicating that they express this marker of migrating neurons. In the regions surrounding the aberrant ventricular cells there also appear to be a greater number of DCX-positive cells that had the morphology of migrating neurons (*i.e*. a stained cell body with a distinct putative leading process, arrows in Fig. 3E, panel 4). These data indicate that a fraction of these aberrant ventricular cells express markers of migratory neurons. Because of this increase in DCX positive cells, neural stem cell homeostasis in *Nfix*-/- mice is currently being investigated.

**Figure 4:**
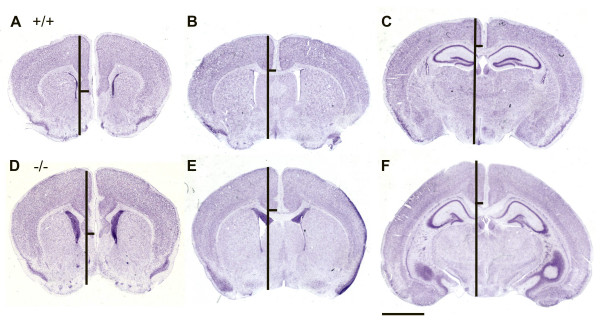
**Expanded DV size of the brain in *Nfix*-/- mice**. Images of Bregma-matched sections of P22 brains of WT (+/+) **(A-C) **and *Nfix*-/- **(D-F) **brains. Brains were fixed in 4% PFA, embedded, sectioned, stained with cresyl violet, and mounted. Three sections of each brain at different Bregma levels were assessed for the DV midline depth of the cortex and the midline DV depth of the entire brain. The vertical bars show the entire DV depth while the horizontal bar denotes the measured depth of the cortex from the dorsal surface. Note the increase in both midline DV cortex and subcortical region depth. Measurements from 3 sections each from 3 pairs of littermate brains of the indicated ages are shown in Table 1. Scale bar = 2 mm.

**Figure 5:**
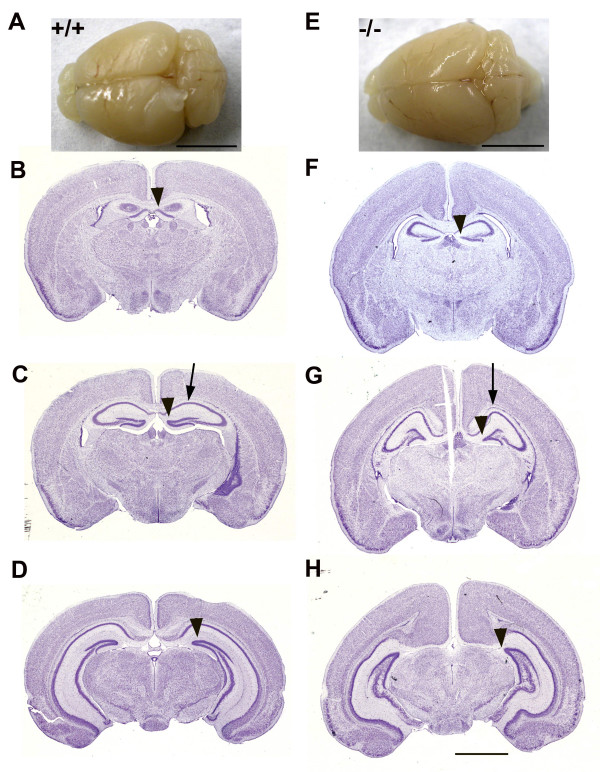
**Aberrant hippocampus and cingulate cortex in *Nfix*-/- mice**. Comparison of the shape of the hippocampus and cingulate cortex in P28 WT +/+ **(A-D) **and *Nfix*-/- **(E-H**) brains. **A) **Image of a dorsal view of the brain of a +/+ mouse. Scale bar = 5 mm. **B**) Coronal section of the +/+ brain at the anterior pole of the hippocampus at about Bregma -0.94. The arrowhead indicates the dentate gyrus. **C) **Coronal section of the +/+ brain at about Bregma -1.70. The arrowhead indicates the dentate gyrus and the arrow the dorsal most point of the hippocampus. **D) **Coronal section of the +/+ brain at about Bregma -2.92. The arrowhead indicates the dentate gyrus. **E) **Image of a dorsal view of the brain of an *Nfix*-/- mouse. Scale bar = 5 mm. Note the difference in A-P extent and the difference in shape of the cerebellum compared to A. **F) **Coronal section of the -/- brain at about Bregma -0.94. The arrowhead indicates the dentate gyrus. **G) **Coronal section of the -/- brain at about Bregma -1.70. The arrow indicates the dorsal enlargement of the hippocampus and the arrowhead the shortened dentate gyrus. **H) **Coronal section of the -/- brain at about -2.92. The arrowhead indicates the medial end of the external capsule. Scale bar = 2 mm for B-D and F-H.

In addition to this aberrant ventricular cell population, the postnatal brains of *Nfix*-/- animals show clear morphological changes. These include a dorsal-ventral expansion of the cortex and the septum and distortion of the hippocampus (Fig. 4). Expansion of the cortex is most easily quantified by comparing the DV height of the cortex in coronal sections as measured from the dorsal brain surface to the most ventral region of the grey matter of the cortex and comparing this to the DV size of the ventral telencephalon and whole brain (Fig. 4). From P22 to P69 both the cortex and septum showed an ~17–20% increase in DV size in *Nfix*-/- versus WT animals (Fig. 4 and Table 1). A similar DV expansion of the cingulate cortex and the septum was seen in postnatal brains from P7 to P69 as assessed by visual examination of serial coronal sections (RMG and JB, unpublished data). This dorsal-ventral expansion of the cortex is consistent with the anterior-posterior cortex expansion seen in gross brain images (for examples see Figs. 3A and 5A &5E). We are currently determining whether the cellular composition of the cingulate cortex is affected by loss of *Nfix*.

**Table 1:** Increased midline DV depth of cortex and subcortical regions in *Nfix*-/- mice.

**Section**	**Age +/+**	**DV ctx mm**	**DV brain mm**	**Diff**	**Age -/-**	**DV ctx mm**	**DV brain mm**	**Diff**	
**1**	**P22**	3.00	5.00	2.00	**P22**	3.50	6.00	2.50	
**2**		1.75	6.00	4.25		2.25	6.25	4.00	
**3**		2.13	6.63	4.50		2.25	7.13	4.88	
**1**	**P45**	4.00	5.38	1.38	**P45**	4.13	6.25	2.13	
**2**		1.88	6.00	4.13		2.38	7.50	5.13	
**3**		1.38	6.75	5.38		2.13	7.25	5.13	
**1**	**P69**	3.75	5.25	1.50	**P69**	4.38	6.75	2.38	
**2**		2.13	5.38	3.25		2.50	6.75	4.25	
**3**		1.25	6.38	5.13		2.00	8.00	6.00	

		2.36	5.86	3.50		2.83	6.88	4.04	**Avg**.
						1.20	1.17	1.15	**Ratio^-/-^/_+/+_**

Triplicate sections similar to and including those shown in Fig. 4 from littermates of the indicated ages were analyzed for the DV depth of the cortex (DV ctx mm) and the DV depth of the entire brain (DV brain mm) and the difference (diff) was used as the DV depth of the septum and ventral region. The average depth of each region was calculated and the ratio of the depths of -/- to +/+ brains was determined. A 15–20% increase in the depth of both the cortex and septum was seen. No standard deviation was calculated because the values are derived from ratios which complicate use of a T-test, but a consistent 15–20% increase in DV depth was seen in multiple pairs of brains.

To ask if the increased cortical size resulted in a more massive brain we assessed the weights of fixed brains at several ages. Consistent with the increase in cortex size, the overall weight of the brains of *Nfix*-/- animals was 11–24% heavier than that of matched littermates (Table 2). This increase in brain weight was seen despite the overall decrease in total body weight seen in the *Nfix*-/- animals. There is also a noticeable trend for the increase in weight to be greater in older compared to younger animals, suggesting an ongoing increase in weight with age. This increase in weight suggests an increase in either neurons or glia and we are in the process of using specific cell markers to assess which cell types are increased in the *Nfix*-/- brains.

**Table 2:** Increase in brain wet weights in *Nfix*-/- mice.

**Age**	**Fixed Brain wt. (g)**		
**Days**	***Nfix*-/-**	***Nfix+/+***	**% incr**.

18	0.506	0.453	11
	0.507		
22	0.513	0.468	11
		0.459	
28	0.602	0.487	24
40	0.548	0.463	18
136	0.647	0.524	23
150	0.608	0.518	17

Brains of littermates of the indicated ages and genotypes were fixed in 4%PFA and weighed. The weights of duplicate brains of the same age and genotype were averaged when possible. Wet weights showed an 11–24% increase at the indicated ages. No standard deviation was determined because the values are derived from a ratio but an increase was consistent in all brains analyzed.

### Aberrant brain development in *Nfix*-/- animals: Hippocampus

Examination of serial coronal sections also demonstrated severe distortion of the hippocampus, particularly evident in sections at the level of the dentate gyrus. At P28 there were several major forebrain abnormalities. Dorsal views (Fig. 5A &5E) of the brains of P28 WT (+/+) (Fig. 5A) and *Nfix*-/- mice (Fig. 5E) show that the overall shape of the two brains is different with the *Nfix*-/- cortex more elongated in the anterior-posterior direction (A-P) compared to the +/+ brain. The extent of the exposed cerebellum is shorter in the *Nfix*-/- brain. As noted above in Fig. 4, the *Nfix*-/- brain is elongated in the dorso-ventral (D-V) direction; (compare Figs. 5B vs. 5F, 5C vs. 5G and 5D vs. 5H). The shape of the corpus callosum at this level is also different; it is more "u-shaped" in the *Nfix*-/- brain (Fig. 5B vs. 5F) and the angle of junction of the fimbria and hippocampus is more oblique in the WT than in the *Nfix*-/- mouse. The shape of the hippocampus is also distorted in the *Nfix*-/- mouse, with a dorsal enlargement in CA1 (Fig. 5C &5G, arrows). The dentate gyrus is shorter in the *Nfix*-/- brains (arrowheads in Fig. 5B &5F, 5C &5G and 5D &5H), and the angle of the "arrowhead" of the gyrus is larger. In addition, in some brains a slightly "wavy" appearance of the CA3/CA4 fields in posterior sections of the hippocampus are seen (*e.g*. Fig. 5H), possibly indicating a change in cytoarchitecture of the pyramidal cell layer here. Finally, the A-P extent of the corpus callosum appears somewhat shorter in the *Nfix*-/- than in the +/+ brain; it has just ended in 5D in the +/+ compared to 5G in the -/- brain. Such a change is consistent with the partial callosal agenesis noted in a previous study [18]; however, as discussed below these data are subject to interpretation due to the acallosal phenotype seen in 129 strain mice.

### *Nfix *expression in developing brain

**Figure 6:**
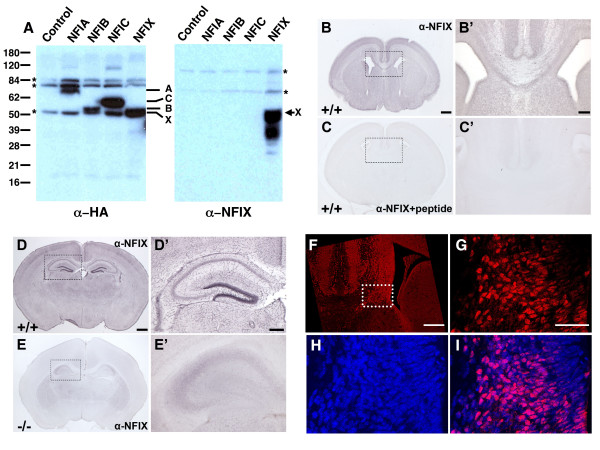
**Specificity of the α-NFIX antisera used for immunohistochemistry**. The specificity of the α-NFIX antibodies was assessed by both Western Blot **(A) **and immunohistochemistry **(B-E')**. **A) **Western blots of extracts containing the indicated NFI proteins. The samples in each lane were as indicated and the left panel shows a membrane probed with α-HA antibodies and the right panel a membrane probed with α-NFIX antibodies. The asterisks to the left and right of the panels denote non-specific bands seen in all lanes, the numbers to the left indicate the size in kDa of marker proteins, the A, B, C and X between the panels shows the migration positions of NFIA, NFIB, NFIC and NFIX respectively as assessed by α-HA staining while the X on the right shows the position of the major NFIX band assessed by α-NFIX staining. The bands below X are most likely proteolysis products lacking the N-terminus since they are not detected by α-HA antibodies. Note no cross reactivity of the α-NFIX antibodies with the other 3 NFI gene products. **B&B') **Coronal section **(B) **and higher magnification **(B') **of a WT E17 brain stained with α-NFIX antibodies. Note clear staining of cortex and subcortical regions. **C&C') **Coronal section **(C) **and higher magnification **(C') **of a WT E17 brain stained with α-NFIX antibodies that had been preincubated with an excess of the peptide to which the antibodies had been generated. **D&D') **Coronal section **(D) **and higher magnification **(D') **of a WT P14 brain stained with α-NFIX antibodies. Note clear cellular staining in the dentate gyrus. **E&E') **Coronal section **(E) **and higher magnification **(E') **of an *Nfix*-/- P12 brain stained with α-NFIX antibodies. Note the absence of cellular staining and only very weak diffuse background staining. **F-I) **Immunofluorescence showing *Nfix *expression in nuclei of cells in wildtype E17 brains. **F) **Low magnification image of coronal section showing *Nfix *expression (red). The section extends from just left of the midline to just past the lateral ventricle on the right and encompasses the right lateral ventricle. Dashed box denotes region expanded in G-I. **G) **High magnification image of immuno-fluorescence of *Nfix *expression. **H) **High magnification image of Dapi staining of nuclei, **I) **Merged images of G and H showing that *Nfix *expression is predominantly nuclear. Bar in B = 400 μm for B&C, bar in B' = 80 μm for B'&C', bar in D = 600 μm for D&E and bar in D' = 250 μm for D'&E', bar in F = 200 μm and bar in G = 50 μm in G-I.

To determine whether the morphological changes seen in *Nfix*-/- brains might be cell-autonomous changes due to loss of *Nfix *expression in neurons or glia, we asked where *Nfix *is expressed during pre- and post-natal brain development. For these studies we performed immunohistochemistry using an NFIX-specific anti-peptide antibody (Fig. 6, α-NFIX). This antibody detects only NFIX and no other NFI family member as assessed by Western blot analysis of cells expressing each of the 4 NFI gene products (Fig. 6A, compare left (α-HA) and right (α-NFIX) sides of panel A). Specificity of the antibody for immunohistochemistry was first tested by demonstrating loss of staining of WT brains upon preincubation of the antibody with the peptide used for its generation (Fig. 6, panels B&B' vs. C&C'). In addition, the antibody detects strong cellular staining of cells in +/+ brains but only extremely faint and diffuse background staining in *Nfix*-/- brains (Fig. 6, panels D&D' (+/+) vs. E&E' (-/-)). By comparing *Nfix *expression using immunofluorescence (Fig. 6F &6G) with DAPI staining in the same sections (Fig. 6H &6I), it appears that NFIX protein expression is predominantly nuclear. Using this specific antibody we assessed NFIX protein expression in WT (+/+) brains.

**Figure 7:**
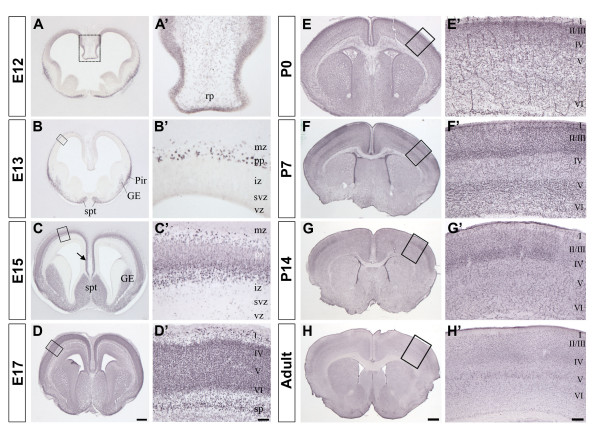
**NFIX expression in the developing telencephalon**. NFIX expression during telencephalon development from E12 to adult. In each panel **(A-H) **the square box indicates a corresponding higher magnification image in **A'-H'**. A' and B' show NFIX expression in the roof plate and the preplate, respectively. Panels C'-H' show the expression of NFIX during cortical layer development. Scale bar in H is 600 μm for panels A and B; 520 μm for panel C; 480 μm for D; 400 μm for E; 625 μm for panels F-H. Scale bar in H' is 150 μm for panel A'; 80 μm for B'; 50 μm for C' and D'; 200 μm for E'; 100 μm for F'; 250 μm for G' and H'. GE, ganglionic eminence; Pir, piriform cortex; spt; septum; iz, intermediate zone; mz, marginal zone; pp, preplate; rp, roof plate; sp, subplate; svz, subventricular zone; vz, ventricular zone; I, II/III, IV, V, VI, cortical layers.

We previously reported that *Nfix *mRNAs were detected in the neocortex at embryonic (E) stage E11.5 [8]. Here we found that the NFIX protein is detected as early as E11 in the preplate and ventricular zone of the dorsal telencephalon (data not shown) as well as in the roof plate and ventricular zone at E12 (Fig. 7A and 7A'). At E13 NFIX protein is present in the preplate, septum, ganglionic eminence and piriform cortex (Fig. 7B and 7B'). By E15 NFIX protein is located in the deeper layers of the cortical plate as well as some scattered cells in the marginal zone (Fig. 7C and 7C'). NFIX is also strongly expressed in the cingulate cortex at E15 (arrow in Fig. 7C). At E17 and P0 all the cortical layers strongly express NFIX (Fig. 7D and 7D') with stronger levels of expression observed in upper layers (layers II and III Fig. 7E and 7E'). NFIX expression is stronger in layers II/III and V (Fig. 7F and 7F') at P7 compared to P0, and remained in layers II/III at P14 (Fig. 7G and 7G') while in the adult brain, only weak immuno-staining was observed (Fig. 7H and 7H'). The analysis of NFIX expression in the developing telencephalon shows that NFIX is highly expressed in the cortex and therefore might be playing an important role during neocortex development. The expanded cortex seen in *Nfix*-/- animals is consistent with this hypothesis.

### *Nfix *expression at the cortical midline

**Figure 8:**
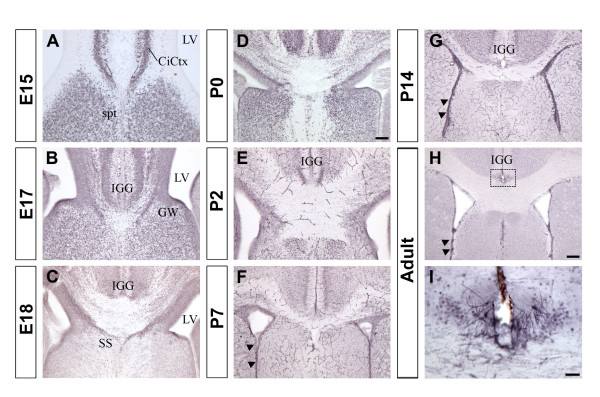
**NFIX expression at the cortical midline**. Expression of NFIX protein at the midline from E15 to adult. Panel **A **shows that NFIX is strongly expressed in the cingulate cortex and the septum at E15. NFIX is detected in the regions containing the indusium griseum (IGG) and the glia wedge (GW) at E17 (panel **B**) and in the region containing the subcallosal sling (SS) at E18 (panel **C**). NFIX was also present at the cortical midline from P0 to adult **(D-I) **and in the ventricular zone from P7 to adult (arrowheads in F-H). Panel **I **(higher power of view of the box in **H**) shows NFIX positive cells in the region of the indusium griseum. Scale bar in D is 70 μm for A; 100 μm for B, C and D. Scale bar in H is 200 μm for panels G and H. Scale bar in I is 30 μm. CiCtx, cingulate cortex; spt; septum; LV, lateral ventricle; IGG, indusium griseum glia; GW, glia wedge; SS, subcallosal sling.

At the cortical midline, four midline glial populations have been previously described; the glia within the indusium griseum (IGG), the glial wedge (GW), the midline zipper glia, and the subcallosal (glial) sling (SS, [13,22,23]). We showed previously that in both *Nfia *and *Nfib *knockout mice those populations were disrupted [13,16], implicating NFIA and NFIB in their development. We therefore investigated the expression of NFIX in regions containing these midline glial populations to determine if NFIX might also be involved in their development. At E15, NFIX is found in the cingulate cortex and in the septum (Fig. 8A) but not in the areas containing the midline glia populations. By E17, NFIX was detected in the IGG, GW and the ventricular zone (Fig. 8B), and by E18 NFIX was expressed in the regions containing all four midline glial populations and in the ventricular zone (Fig. 8C). During postnatal development, NFIX proteins are still detected at the midline (Fig. 8D–I) as well as in cells lining the lateral ventricle (aka ependymal cells, arrowheads in Fig. 8F–H). NFIX expression in the regions containing these midline glial structures throughout development indicates a possible role for NFIX in their development. Double-labeling experiments with glial markers and α-NFIX will be required to assess the relative expression of NFIX in glial cells and neurons in these regions. Postnatal expression of NFIX in ventricular cells may be related to the expansion of aberrant ventricular cells in *Nfix*-/- mice.

### *Nfix *expression during hippocampal development

**Figure 9:**
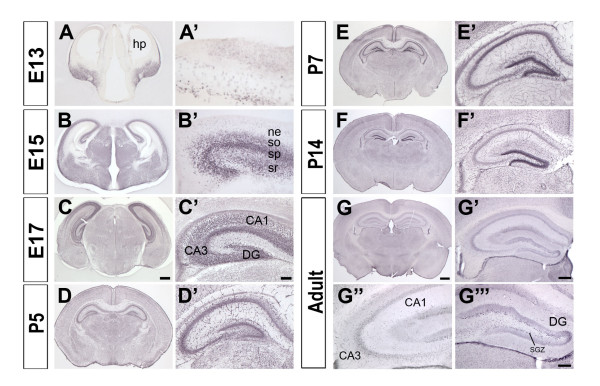
**NFIX expression in the hippocampus**. NFIX protein expression during hippocampal development from E13 to adult. Each panel **(A-G) **was magnified for a better view of the hippocampus **(A'-G')**. NFIX was first expressed in the hippocampal primordium at E13 **(A and A')**. At E15, NFIX expression was found in the stratum oriens, stratum pyramidale and stratum radiatum layers of the hippocampus **(B')**. NFIX was strongly expressed in all the areas of the hippocampus at E17 **(C') **and at P5 **(D')**. At P7 and P14 strong expression of NFIX is detected in the dentate gyrus (**E' **and **F'**, respectively). In the adult (**G **and **G'**) NFIX is expressed at lower levels, although stronger expression is observed in the CA3 region compared to CA1 **(G") **and some scattered cells were detected in the dentate gyrus and the subgranular zone **(G"')**. Scale bar in C is 270 μm for panel A; 360 μm for B; 400 μm for C; scale bar in C' is 60 μm for A', B'; 90 μm for C'; Scale bar in G is 600 μm for D; 625 μm for E, 700 μm for F and G. Scale bar in G' is 200 μm for D'; 260 μm for E', 290 μm for F' and G'. Scale bar in G"' is 100 μm for G"' and G". hp, hippocampal primordium; ne, neuroepithelium; so, stratum oriens; sp, stratum pyramidale; sr, stratum radiatum; DG, dentate gyrus; CA, Ammon's horn; SGZ, subgranular zone.

The aberrant hippocampal development seen in *Nfix*-/- mice prompted us to examine NFIX expression in this region. NFIX proteins are detected as soon as the hippocampal primordium appears at E13 (Fig. 9A and 9A') and expression continues in all layers through E15 (Fig. 9B and 9B'). By E17 NFIX proteins are no longer detected in the stratum radiatum (Fig. 9C and 9C') but become more highly expressed in the differentiating layers of the hippocampus. In the first postnatal week, NFIX expression is observed in the stratum pyramidale (Fig. 9D and 9D') and expression in the dentate gyrus became stronger during postnatal development (at P7, Fig. 9E and 9E'; and at P14 Fig. 9F and 9F') but decreased overall in the adult (Fig. 9G and 9G'). In adult brains, stronger NFIX expression was observed in CA3 compared to CA1 (Fig. 9G") and a few NFIX positive cells remained in the subgranular zone of the dentate gyrus (Fig. 9G"'). Hippocampal expression of NFIX, together with the defects seen in *Nfix*-/- brains indicates a major role for NFIX in hippocampal development.

## Discussion

These data indicate that *Nfix*, like *Nfia *and *Nfib*, plays a major role in brain development. In addition, the poor weight gain and delayed eye and ear opening (Fig. 2) indicate that *Nfix *may play an important role in the development of the gut and possibly the epithelial components of other organs. While this manuscript was in preparation another group generated and characterized mice containing a non-conditional *lacZ*-tagged *Nfix *disrupted allele [18]. Since there are both similarities and differences between the phenotypes of these two distinct mouse mutants we will discuss these in detail below.

The delayed eye and ear opening, poor weight gain, and lethality by ~P22 of *Nfix*-/- animals (Figs. 2A, C–D) are the most striking and earliest visible phenotypes due to loss of *Nfix*. We observed that supplementing the diets of *Nfix*-/- animals with a soft dough supplement helped alleviate the runting of the animals and allowed survival of most animals past P28 (Fig. 2B). Such supplementation had previously allowed us to rescue runting due to tooth defects in *Nfic*-/- mice [17]. However, we have seen no tooth defects in *Nfix*-/- mice. Thus, this partial rescue of the lethality and runting with dietary supplement points strongly towards either a neurological defect that affects eating or digestion, or a defect or delay in gut development in the *Nfix*-/- animals. Our preliminary data (not shown) indicate some changes in the timing of gene expression patterns in the small intestine. While the diet-supplemented *Nfix*-/- animals still have lower weights than their heterozygous or WT littermates (Fig. 2B), the weight reduction is far less severe than is seen in non-supplemented animals and ~65% of diet-supplemented *Nfix*-/- mice survive past P40 and ~10% past P90. In one case, an *Nfix*-/- male has sired multiple litters, showing that *Nfix *is not essential for male fertility. A single adult *Nfix*-/- female has given birth to a small litter but failed to nurture it, resulting in death of the pups. Thus it appears that *Nfix *is not essential for reproduction *per se*, but may affect rearing behavior. The leg clasping phenotype seen previously in *Nfix *targeted mice [18] was also seen in our *Nfix*-/- animals maintained on soft dough, indicating that the neuroanatomical defects described below likely have behavioral consequences independent of the nutritional status of the animals. It will be of great interest to determine whether the observed failure to thrive is due to direct defects or delays in intestine development or function, or whether the neuroanatomical defects discussed below could influence food sensing or eating behavior. To distinguish between these possibilities it will be necessary to perform detailed analysis of intestine development and function, careful behavioral studies on the mutant mice, and to use the conditional allele described here to delete *Nfix *solely from brain or intestine cells and determine which region is responsible for the phenotype.

### Brain developmental changes in *Nfix-/-* mice

Previous studies showed that both *Nfia *and *Nfib *are essential for normal brain development [12,13,16]. Loss of either gene results in agenesis of the corpus callosum although it is unclear whether the mechanisms leading to this loss are identical in the two mutants. Loss of *Nfia *also results in postnatal hydrocephalus in mice, while the P0 lethality seen in *Nfib*-/- mice precludes analysis of postnatal phenotypes in these animals. The changes seen in *Nfix*-/- brains are quite distinct from those seen with loss of either *Nfia *or *Nfib*. For example, callosal axons cross the midline in *Nfix*-/- animals (Figs. 3B–C, 4B &4E, 5B &5F). This differs markedly from the complete callosal agenesis seen in homozygous *Nfia *and *Nfib *knockouts. In a recently published study of a non-conditional *Nfix *disruption the authors conclude that loss of *Nfix *results in both hydrocephalus and partial callosal agenesis [18]. One possible explanation for this partial agenesis of the corpus callosum is the observation that virtually all 129 strain mice contain a recessive gene that results in callosal agenesis in ~25% of offspring and a reduced callosum size in 50% of progeny [24]. To allow robust analysis of callosal defects, all mouse knockouts derived from 129 strain ES cells should be backcrossed at least 10 generations onto a strain that does not carry this trait (*e.g*. C57BL/6). Thus it will be important to determine whether callosal defects are evident when the *Nfix*-/- mice are backcrossed to C57BL/6 for 10 or more generations.

Unlike the previous report, we have not detected hydrocephalus in our *Nfix*-/- mice, but instead find unusual *Pax6*- and DCX-positive cells within the lateral ventricles of the mice (Figs. 3C–E). These aberrant cells are present from at least P0 until past P69 although their abundance appears to decrease somewhat with age. These cells have been detected in every *Nfix*-/- brain sectioned (N = 10) and in no WT brains. While the nature and source of these cells is as yet unknown, it is intriguing that at P12–16 they express *Pax6*, a marker for neural progenitor cells [19,20]. The normal expression of *Nfix *in the ventricular zone of postnatal animals at this age (Figs. 7F &7G, 8F–H), together with the knowledge that ventricular zone progenitor cells express *Pax6*, strongly suggests that these cells are aberrant ventricular zone cells and may represent a hyperproliferation of these potential neural progenitor cells. Of possible relevance to this change in ventricular cell number, *Nfix *had previously been implicated in the etiology of glioblastoma formation in a mouse model system [25,26]. *Nfix *is a recurrent integration target in glioblastomas generated by intraventricular injection of retroviruses carrying the PDGF-B chain [25]. This system is used to identify oncogenes or tumor suppressor genes that act in concert with PDGF-B chain to generate glioblastomas. Interestingly, *Nfia*, *Nfib *and *Nfic *were also shown to be integration targets in this system [25]. Thus it will be of great interest to determine whether *Nfix*-/- mice are more prone to glioblastoma formation induced by PDGF-B retroviruses and other agents, and whether double mutants of *Nfix *with *Nfia*, *Nfib *or *Nfic *have additional neurological or oncological phenotypes. It is unclear why the previous study did not note these aberrant ventricular zone cells, since they have been seen in 100% of the *Nfix*-/- brains from P0–P69 (N = 10). Given that the phenotype is seen in 100% of the homozygous Cre-deleted animals, but not in any heterozygous or homozygous conditional allele-containing animals, this phenotype cannot be due to the presence of a linked or unlinked secondary mutation.

It is of interest that a fraction of these aberrant ventricular cells also express DCX, a marker of migratory neurons [21]. The presence of this marker could indicate the differentiation of aberrant ventricular zone cells into migratory neurons. However, an alternative explanation is that these aberrant ventricular cells represent migratory neurons destined normally for the rostral migratory stream (RMS), which fail to follow their normal migratory route to the olfactory bulb and instead populate the ventricle. In this case, these cells would be exhibiting a cell migration defect that could be related to the axonal guidance defects that cause callosal agenesis in *Nfia *and *Nfib *KO mice. Were the phenotype due to a failure of ventricular zone cells to enter the RMS, one might expect a decrease in the size of the olfactory bulb in the mutants as has been seen with some other mutations affecting cell migration or survival [27-29]. While gross examination and preliminary data from sections of the olfactory bulb do not show major changes of structure as have been seen with some mutations affecting RMS migration [27], careful birthdating, cell marking, and migration studies must be performed to determine whether these aberrant ventricular cells are indeed migratory neurons. Should defects in olfactory bulb structure or function be detected, they could contribute to feeding defects in the animals and the observed failure to thrive.

Our current working hypothesis is that the expanded cortex (Figs. 3, 4, 5), and the distortion of the hippocampus (Fig. 5), may be directly related to the process that produces these aberrant ventricular zone cells. Both the cortical expansion and aberrant ventricular cells have been seen in 100% of *Nfix*-/- animals (N = 10) in multiple sections along the anterior-posterior axis. Similar or even more dramatic expansions of the cortical surface area have previously been detected due to increased β-catenin signaling [30] or by deletion of the pro-apoptotic caspase, *Casp9 *[31]. While the cortical expansion seen here is less than that seen with these other mutations, it will be of interest to determine whether either of these pathways may be affected by loss of *Nfix*. In particular, it will be important to determine whether the cortical expansion is consistent with recent models of mechanisms regulating cortex development [32]. If the aberrant ventricular cells detected here indeed represent an increase in the neural stem cell population, it is possible that continued slow proliferation and differentiation of such stem cells could both expand the cortex and cause distortions of the hippocampus due to excessive cell production. We will test this hypothesis with cell-marking and birthdating studies and assessing neural stem cell numbers in WT and *Nfix*-/- animals.

### Possible differences in phenotype compared with previous studies

As noted above, previous investigators found postnatal lethality, partial callosal agenesis, severe hydrocephalus and skeletal defects in *Nfix*-/- mice which were either not seen or were significantly less severe in this study. Both studies deleted exon 2 of *Nfix *which as discussed above should generate a similar null allele. Some possible reasons for these apparent phenotype differences include mouse strain differences, nutritional differences, and subtle differences in the disrupted allele, each of which is discussed below. Different ES cells lines were used in the two studies and the mice analyzed were at differences stages of back-crossing to the C57BL/6 background. In the current study, animals were backcrossed from 3–5 generations to C57BL/6 and thus should contain predominantly C57BL/6 genetic alleles. As discussed above, callosal agenesis is a known phenotype of 129 strain mice and it is necessary to backcross to a strain with normal callosum formation (*e.g*. C57BL/6) before assessing callosum formation. Hydrocephalus is also sometimes associated with callosal agenesis and thus these two apparent phenotypic differences may be linked. Any differences in these phenotypes between the two KO strains should diminish with further backcrossing if strain differences are significant.

In the current study, we were able to rescue substantially the postnatal lethality of the *Nfix*-/- mice by supplementation with a soft dough diet. Without this supplementation, all *Nfix*-/- animals lost over half their body weight from P10–P22 and died, apparently of starvation. The previous study examined animals that were in the process of severe malnutrition and it is possible that such malnutrition could contribute to both hydrocephalus and skeletal defects. Given the normal cranial bones seen in our *Nfix*-/- animals (not shown), we feel it is unlikely that bone defects contribute in any way to the brain malformations seen here. It will be necessary to rear the two KO strains under identical nutritional conditions to assess whether this is a true difference in phenotype. Finally, the two strains differ in the structure of the final *Nfix *KO allele. The earlier KO allele, after Cre deletion of the neomycin-resistance marker, contained a fusion of a *lacZ *coding region to the first 4 coding residues of the 2nd exon, a deletion of the remainder of the 2nd exon and 32 residues of the adjacent intron, and addition of a loxP site 3' to the *lacZ *coding region. The current study generated a conditional allele with an insertion of a floxed 2nd exon and a neomycin-resistance gene within the adjacent 3' intron. This conditional allele was then bred to a ZP3-Cre transgenic strain resulting in deletion of the 2nd exon, ~1 kb of intron sequence 5' and 3' to exon 2 and the neomycin resistance marker, and retention of only a single loxP site. Thus the previous allele deleted all but 4 residues of exon 2 and some adjacent intron sequence and contains a *lacZ *coding region and loxP site, while the current allele deletes all of exon 2 and some adjacent intron sequence and contains a single loxP site. Thus it is possible that either the addition of the *lacZ *coding region or differences in the amount of intron sequence deleted between the two KO alleles could affect either the expression of adjacent genes or possible expression of downstream exons of either *Nfix *KO allele. The presence of termination codons at the 3' end of the *lacZ *sequence of the previous allele and early within the out-of-frame spliced exon1-exon 3 transcript of the current KO allele significantly reduces the probability of any translation of downstream exons from either locus. However it is possible that aberrant internal initiation of translation from exons downstream of the deleted exon 2 could occur with either allele. Since such unusual downstream internal initiation could occur theoretically at any initiation codon downstream of exon 2, to formerly test this hypothesis it would be necessary to generate antibodies to all of the potential in- and out-of-frame coding regions downstream of exon 2 to test for the presence of such peptides. This would be particularly difficult given the known alternative splicing of *Nfix *transcripts downstream of exon 2. Since all previous studies indicate that exon 2 codes for the DNA-binding and dimerization domains of NFI proteins, the current evidence makes it unlikely but not impossible that such aberrant downstream peptides could mediate some of the apparent differences in phenotype seen between the current and previous KO strains. We would reiterate that the lack of phenotype seen in the current conditional *Nfix *allele, combined with the highly penetrant phenotype of the Cre-deleted KO allele, strongly indicates that there are no unlinked or linked mutations that contribute to the observed phenotype in the current KO allele. Additional studies may be needed to reconcile the apparent differences seen between the previous and current *Nfix *KO alleles.

It has been noted that the temporal expression pattern seen here for NFIX protein in developing cortex may differ somewhat from the pattern seen previously for *Nfix *mRNA [8]. In previous studies *Nfix *mRNA was clearly detected in the dorsal VZ/SVZ region by E11.5 while here only weak expression of NFIX protein is seen in these regions at E12 and E13, with robust expression being seen at E17. Such differences may be due to differential translation of *Nfix *mRNA at these times, or to differences in the sensitivity and cellular resolution of *in situ *mRNA detection versus immunostaining. We are currently examining in more detail both the *Nfix *mRNA and protein expression patterns from E12–E17 to resolve these possible discrepancies.

Given the high degree of sequence homology among NFI family members and the overlapping patterns of expression of the 4 mouse NFI genes, it is perhaps surprising that each of the mouse genes has been shown to have distinct roles in development. However, we are now beginning to see some similarities and possibly shared functions among the 4 mouse NFI genes. Loss of *Nfia *results in hydrocephalus, agenesis of the corpus callosum, defects in midline structures and changes in gene expression associated with a delay in oligodendrocyte maturation [12,13,33]. Many of these defects could be related to altered glial cell development in the brain. Similarly, loss of *Nfib *is also associated with midline glial cell defects and agenesis of the corpus callosum [16]. The recent observation that either *Nfia *or *Nfib *can promote gliogenesis in the developing spinal cord also points to potential sharing of function during development [14]. We are currently testing for direct evidence of overlapping or shared functions between the 4 NFI genes by generating double-mutant mice in specific pairs of NFI genes.

Together with previous studies, these data indicate that 3 of the 4 NFI genes (*Nfia*, *Nfib *and *Nfix*) play important roles in brain development. In addition to their functions in brain development, at least 3 of the NFI genes also have essential roles in other organ systems including lung (*Nfib*) [16], tooth (*Nfic*) [17] and intestine (*Nfix*). Since products of all 4 NFI genes appear to bind to the same DNA sequences with similar affinities, it will be of great interest to determine whether there are shared or distinct target genes for the different NFI isoforms in tissues affected by loss of individual NFI genes.

## Methods

### Gene targeting and mouse strains

Genomic clones encompassing *Nfix *exon 2 were isolated from a mouse 129/Sv phage library as described previously [12,17]. A targeting vector was constructed with a 4.2 kb 5' homology arm containing all of exon 2 and 633 bp of intron 2, with a loxP site inserted 406 bp 5' to the start of exon 2. The 5' arm was followed by an FRT-flanked PGK-neo expression cassette in the opposite transcriptional orientation, a 3' loxP site, the contiguous 2.7 kb 3' homology arm and a PGK-diphtheria toxin A chain cassette in the opposite transcriptional orientation. Cre-mediated recombination deletes all of exon 2, 406 bp of intron 1 and 633 bp of intron 2. The vector was electroporated into J1 ES cells (129S4, [34]), G418-resistant colonies were picked, expanded and banked until PCR screening was completed. DNA from ES cell clones was isolated and subjected to nested PCR using pairs of primers with one primer located outside of the 5' and 3' ends of the targeting vector and the other primer located within the 5' and 3' lox P sites (see Fig. 1 for details). 4 of 361 colonies showed correct targeting at the 5' and 3' ends and clone 38 was injected into C57Bl/6 blastocysts to generate chimeric animals. Chimeras were bred with C57Bl/6 females and agouti progeny were screened for the presence of the targeted conditional KO allele by PCR with primers flanking the 5' loxP site. To generate the Cre-deleted knockout allele (KO), males containing one or two Conditional Alleles (CA) were bred with females expressing Cre recombinase in their oocytes from a ZP3-Cre transgene [35]. Progeny were screened for Cre-mediated loss of exon 2 by PCR with a primer 5' to the 5' loxP site and 3' to the 3' loxP site of the Conditional Allele. Male mice heterozygous for the Conditional Allele or the Knockout Allele were backcrossed to C57Bl/6 mice and to heterozygous females to generate homozygous progeny. All experiments were performed on a mixed 129S4/C57Bl/6 background and mice backcrossed for 2–5 generations to C57Bl/6 have indistinguishable phenotypes.

### Analysis of animal and brain weights and gross morphology

Animals were weighed from postnatal day 6 (P6). Animals were observed visually daily from postnatal day 1 (P1) and the days at which the eyelids and ear canals opened were noted. The times shown are for complete eye and ear canal opening. Brain weights were assessed on brains fixed in 4% PFA at the ages indicated.

### PCR and QPCR transcript analysis

Tissues were extracted immediately following dissection with TRIzol (Invitrogen), total RNA was isolated and cDNA was generated from 5 μg RNA with Superscript II reverse transcriptase (Invitrogen) and random primers as recommended by the manufacturer. *Nfix *transcripts were analyzed by PCR using an exon 1-based sense primer and an exon 3-based anti-sense primer and products were resolved on 2.5% agarose gels. Transcript levels of all genes analyzed were quantified by QPCR with a Bio-Rad iCycler real-time PCR machine using gene-specific primers that span multiple exons as described previously [17]. Sequences of the primers are available upon request.

### Brain sectioning

Embryonic mouse brains were collected from embryonic day (E) E12 to P0. Prior to E15, brains were drop-fixed in 4% paraformaldehyde in PBS. From E15 onwards mice were perfused transcardially with saline followed by 4% paraformaldehyde in PBS. For Figures 6, 7, 8, 9, brains were sectioned at 45–50 μm using a vibratome (Leica, Deerfield, IL). For experiments shown in Figures 3, 4, 5, mice were sacrificed by decapitation under Nembutal anesthesia and the brains of postnatal day 7 (P7) to adult *Nfix*-/- and *Nfix*+/+ mice were excised, fixed, embedded, sectioned and stained using standard procedures. Brains were fixed overnight in 10% buffered-formalin or 4% paraformaldehyde in PBS and then cryoprotected in fixative with 15% then 30% sucrose and stored at 4°C. Some brains were embedded in 15% gelatin/5% albumin matrix and equilibrated in 30% sucrose in fixative. 40 μm frozen sections were cut on a sliding microtome or a cryostat. Sections were stored in fixative at 4°C or in a cryoprotection solution of glycerol/ethylene glycol at -20°C. Sections were mounted on gelatin coated slides and stained with cresyl violet. Some animals were subjected to deep anesthesia and transcardial perfusion with saline followed by 4% paraformaldehyde in PBS. The brains were removed and postfixed in 4% paraformaldehyde for 1–3 days, cryoprotected in sucrose in PBS and sectioned and stained as shown above.

### Immunohistochemistry

NFIX expression in brain sections was analyzed using a rabbit polyclonal anti-NFIX serum raised against amino acids 277–291 of human NFIX made by Geneka Biotechnology Inc (cat# 16021118, Montreal, Canada) and now available from Active Motif (cat# 39072, Carlsbad, CA, USA). Other antibodies used were as follows: Pax6 *(*Chemicon, rabbit), doublecortin (DCX, Santa Cruz sc-8606, goat) and phospho-histone H3 (pH3, Upstate, rabbit). Sections were washed with 1X phosphate buffered saline (PBS) and incubated for 2 hours with a blocking buffer containing 2% goat serum (v/v, S-1000, Vector Laboratories, Burlingame, CA) and 0.2% Triton X-100 (v/v; Sigma, St. Louis, MO) in 1X PBS. Anti-NFIX was used at 1/20,000 for embryonic tissues and 1/12,500 for postnatal tissues, anti-*Pax6 *was used at 1/25,000, anti-DCX was used at 1/500 and anti-pH3 was used at 1/2000. Sections were incubated overnight at room temperature then washed several times in PBS and incubated with a biotinylated secondary antibody (Vector Laboratories, Burlingame, CA) for 1 to 2 hours at room temperature, followed by processing with a VECTASTAIN ABC kit for 1 hour at room temperature (A used at 1/500, B used at 1/500, PK6100, Vector Laboratories). Sections were processed for color reaction using nickel- 3,3'-Diaminobenzidine (DAB, D5905, Sigma) solution (2.5% nickel sulfate and 0.02% DAB in 0.175 M sodium acetate) activated with 0.01% (v/v) hydrogen peroxide or the glucose-oxidase modification thereof [36], washed with 1X PBS multiple times, mounted on 3% gelatin-coated slides and coverslipped with DPX mounting medium (Electron Microscopy Sciences, PA). Immunofluorescent staining was performed using a Cy3-labeled goat anti-rabbit secondary antibody and DAPI (300 nM) staining of nuclei. Images were acquired on a PowerPhase digital camera (PhaseOne, Coppenhagen, Denmark), Zeiss AxioCam HRc (Zeiss, Germany) or a Zeiss LSM 510 Meta confocal microscope and were processed using Adobe Photoshop.

### Western Blot analysis

Extracts of JEG3 choriocarcinoma cells transfected with vectors expressing N-terminal HA-tagged versions of NFIA, NFIB, NFIC and NFIX were subjected to SDS-polyacrylamide gel electrophoresis and Western blot analysis as described previously [8]. Duplicate gels containing the samples indicated were blotted to PVDF membranes, probed with antibodies against the HA-tag (Roche, C125A) or NFIX (Active Motif, described above) and subject to chemiluminescent detection (ECL, Amersham).

## Authors' contributions

CEC generated the targeting vector and participated in mouse dissections, RNA preparation and manuscript production, Y-TY screened ES cells for targeted integration and did the initial sectioning of mouse brains, JSB participated in sectioning and staining of mouse brains, analysis of brain morphology, DCX immunohistochemistry and manuscript production, JMO performed maintenance and breeding of mice, mouse dissections, tissue and RNA isolation and QPCR analyses of gene expression and discovered that soft dough could rescue the lethality of *Nfix*-/- mice, CP discovered that the aberrant ventricular cells expressed *Pax6*, assessed NFIX expression in developing brain and aided in manuscript production, MP verified in multiple independent mice the expression of *Pax6 *in the aberrant ventricular cells, pH3 immunohistochemistry, and aided in manuscript production, EDL aided in generation of the *Nfix*KO ES cells and manuscript preparation, LJR supervised CP and MP and participated in manuscript preparation, interpretation of NFIX expression patterns and experiment planning and RMG initiated and directed the overall project, designed the PCR primers for assessing targeted integration and genotype, directed Y-TY, participated in some mouse dissections and did most of the manuscript preparation.

## List of abbreviations used

DCX: doublecortin; DV: dorsal-ventral; NFI: Nuclear Factor I; *Nfia*: Nuclear Factor I A gene; *Nfib*: Nuclear Factor I B gene; *Nfic*: Nuclear Factor I C gene; *Nfix*: Nuclear Factor I X gene; NFIX: Nuclear Factor I X protein; PBS: phosphate buffered saline.
